# Bone Marrow Stromal Cells Alleviate Secondary Damage in the Substantia Nigra After Focal Cerebral Infarction in Rats

**DOI:** 10.3389/fncel.2019.00338

**Published:** 2019-07-24

**Authors:** Jizi Jin, Yanyan Tang, Kongping Li, Xialin Zuo, Lixuan Zhan, Weiwen Sun, En Xu

**Affiliations:** ^1^Department of Neurology, Institute of Neurosciences, The Second Affiliated Hospital of Guangzhou Medical University, Guangzhou, China; ^2^Key Laboratory of Neurogenetics and Channelopathies of Guangdong Province, Ministry of Education of China, Collaborative Innovation Center for Neurogenetics and Channelopathies, Guangzhou, China

**Keywords:** cerebral infarction, substantia nigra, bone marrow stromal cells, secondary degeneration, neurorestoration

## Abstract

Transplantation of bone marrow stromal cells (BMSCs) is a promising therapy for ischemic stroke. Previously, we had reported that the secondary degeneration occurred in the ipsilateral substantia nigra (SN) after permanent distal branch of middle cerebral artery occlusion (dMCAO) in Sprague-Dawley rats. However, whether BMSCs have neurorestorative effects on the secondary damage in the SN after focal cerebral infarction has not known. In this study, rats were subjected to dMCAO followed by intravenous administration of BMSCs 1 day later. We found that transplanted BMSCs survived and migrated to cortical infarct areas and ipsilateral SN. Furthermore, BMSCs promoted neurogenesis through proliferation and differentiation in the SN after dMCAO. Rats implanted with BMSCs showed significant improvement in their performance of modified neurological severity scores and adhesive-removal test. Engrafted BMSCs enhanced survival of dopaminergic neuron, reduced gliosis in the ipsilateral SN, and increased contents of dopamine (DA) and its metabolites in the ipsilateral striatum after dMCAO. With pseudorabies virus-152 as a retrograde tracer, we also demonstrated that BMSCs could effectively enhance the cortico-striatum-nigral connections. These results suggest that BMSCs transplantation exerts neurorestorative effects after cortical infarction through promoting endogenous neurogenesis, increasing contents of DA and its metabolites, alleviating the secondary neuronal damage in the SN, enhancing the cortico-striatum-nigral projections pathway, and finally improving the neurological functional outcome.

## Introduction

Stroke has been the leading cause of mortality and disability in China, the current mortality rate in China is 157 per 100,000, constituting almost 1/3 of the total number of deaths from stroke worldwide (Liu et al., 2011; Wang et al., 2017). Among the survivors about 15–30% left with permanent disability (Go et al., 2014). It has been accepted that cortical cerebral infarction leads to neuropathologic damages not only at primary lesion site, but also in nonischemic remote regions such as hippocampus, thalamus, substantia nigra (SN), distal pyramidal tract, peripheral nerves and muscles (Forno, 1983; Zhang et al., 2012; Dang et al., 2016; Zuo et al., 2016, 2018). This phenomenon is termed as post-stroke secondary degeneration. Focal cerebral infarction leads to dynamic trans-neuronal degeneration in non-ischemic remote brain regions, with the disruption of connections to synapsed neurons sustaining ischemic insults (Zuo et al., 2018). Owing to SN has extensive efferent and afferent fibers connecting with globus pallidus, ventrolateral nucleus and motor cortex, SN is a very common brain structure that is subjected to secondary degeneration after distal middle cerebral artery occlusion (dMCAO). On the other hand, SN is composed of dopaminergic neurons. It has been reported that the secondary damage in the SN after focal cerebral infarction was associated with the development of vascular Parkinson’s syndrome, sustained dementia, and poor neurofunctional outcomes (Kirton et al., 2007; Jellinger, 2008; Domi et al., 2009; DeVetten et al., 2010; Rodriguez-Grande et al., 2013). Secondary degeneration of SN and the corticospinal tract in patients was demonstrated as a hyperintensity lesion within 1–4 weeks after MCA infarction on diffusion-weighted imaging or fluid-attenuated inversion recovery and T2-weighted MRI (Nakajima et al., 2010; Ohe et al., 2013). Also, the observation in monkeys from Chen et al. (2015) revealed an area of high signal in the ipsilateral SN on T2-weighted MRI at 1 week after occlusion in the distal M1 branch of MCA by electrocoagulation. Previously, we have demonstrated that secondary damage occurred in the SN after focal cortical infarction (Zuo et al., 2018). Although some acute phase therapies such as intravenous recombinant tissue plasminogen activator (rt-PA) and endovascular treatment have been shown to improve ischemic stroke outcome, these treatments are only available for a limited number of patients because of the short time window (Campbell et al., 2018; Grossberg et al., 2018). Hence, alleviating secondary neurodegeneration can be a promising target of neuroprotection and neurorestoration beyond the therapeutic time window for acute stroke.

Over the past two decades, bone marrow stromal cells (BMSCs) based neurorepair has emerged as a promising therapeutic strategy for ischemic stroke. As a class of unique, self-renewing cells, BMSCs give rise to differentiated progeny when implanted into appropriate tissues (Maria Ferri et al., 2016). The experimental studies in animal stroke models have shown that the transplantation of BMSCs improved neurofunctional outcome through cellular replacement, stimulating endogenous neurogenesis and angiogenesis, modulating inflammatory environment and reducing the formation of glial scar (Panepucci et al., 2004; Shen et al., 2006; Gutiérrez-Fernández et al., 2013; Fukuda et al., 2015).

It is well known that BMSCs can proliferate and migrate in the brain (Yano et al., 2005; Feng et al., 2017). However, there is no information on the roles of BMSCs affecting neurorestoration in the secondary nigral degeneration after cortical ischemic stroke. Thus, the present study aims to investigate whether BMSCs injected from tail vein enters the brain, migrates to the SN, and attenuates the secondary nigral degeneration following dMCAO. We also sought to examine the possible mechanism of BMSCs on the secondary nigral degeneration after focal cerebral infarction in adult rats.

## Materials and Methods

Adult, male Sprague-Dawley (SD) rats, weighing 280–320 g (10–12 weeks old) were obtained from Southern Medical University (Guangzhou, China). Rats used in the experiment were housed in a controlled environment under standard temperature (22 ± 1°C) and a 12 h light/dark cycle with free access to food and water. Weight gain and health condition of rats are comparable between different groups. All animal procedures were performed in accordance with Animal Research: Reporting *in vivo* Experiments guidelines and were approved and monitored by the Animal Care and Use Committee of Guangzhou Medical University (Guangzhou, China). All efforts had been made to minimize the suffering of animals and the number of animals used.

In this study, 189 rats were used for the experiments. One rat during the surgical procedure and 2 after surgery died in the dMCAO groups. Three rats in the vehicle groups and 1 in the BMSCs groups died during the surgical procedure. In addition, 2 rats died after injection of pseudorabies virus (PRV)-152 in the ipsilateral SNr, and 5 were excluded because neither neurologic deficit nor cortical infarction after dMCAO was observed.

### Animal Model

Permanent occlusion of distal branch of middle cerebral artery was performed using an electrocoagulation methodology described previously (Tamura et al., 1981; Xing et al., 2012). In brief, rats were placed in the anesthesia induction box supplied with 3–4% isoflurane at 3 L/min in 100% oxygen. Anesthesia was maintained with 1.5–2.5% isoflurane at 800 mL/min in 100% oxygen, delivered through a nose mask (SurgiVet, Waukesha, WI, United States) during the surgical procedure. The distal striatal branch of MCA was exposed and occluded by unipolar electrocoagulation under an operating microscope. Rectal temperature of the animals was monitored and maintained at approximately 37°C throughout the procedure. Sham-operated animals were performed with the same surgical procedures except for the electrocoagulation of dMCAO. After surgery, the rats were allowed to wake up and evaluated the neurological status as described by previously (Rodriguez-Grande et al., 2013). Rats neither with neurologic deficit nor cortical infarction were excluded from this study.

### Histology

Animals were intracardially perfused with normal saline, followed by 4% paraformaldehyde (PFA) in phosphate buffer saline (PBS) (0.01 M, pH 7.4) under anesthesia with 10% chloral hydrated administered intraperitoneally (350 mg/Kg). The brains were pos-fixed for 12 h in 4% PFA and then cryoprotected with 10, 20, 30% sucrose in the same fixative overnight. Coronal tissue blocks (bregma 1.7 mm to −5.8 mm) were cut on a freezing microtome (Leica CM1950, Heidelberg, Germany) into 30 μm-thick sections. According to the standard procedure, Nissl staining was performed with 0.1% cresyl violet (MilliporeSigma, Burlington, MA, United States), and then sections were dehydrated with 90 and 100% ethanol and immersed into dimethylbenzene.

Nissl staining was used for infarct volume evaluation. Relative infarct volume was expressed as the percentage of the contralateral hemisphere (Swanson et al., 1990). Briefly, the coronal brain sections from bregma level of 1.0 mm to −1.0 mm were analyzed. Relative infarct volumes at 28 days after dMCAO were evaluated from the contralateral hemisphere volume (Vc) and ipsilateral nonischemic hemisphere volume (Vi) according to the equation (Vc-Vi)/Vc × 100%.

### Immunohistochemistry

The rats were divided into four groups, sham-operated, dMCAO, vehicle and BMSCs groups for immunohistochemical experiments. Rats were euthanized at 7, 14, and 28 days after dMCAO, respectively (*n* = 7 in each group). Single-label immunohistochemistry was conducted by the avidin-biotin complex (ABC) peroxidase method (Zhan et al., 2010). Briefly, sections from bregma −4.52 to −6.3 mm were rinsed with 0.01 M PBS, treated with 3% H_2_O_2_ for 30 min, followed by 5% normal serum for 1 h at room temperature, and then incubated overnight at 4°C with primary antibodies, including rabbit polyclonal anti-nuclear-associated antigen Ki-67 (Ki-67) (1:2000, Abcam, Cat# ab15580, RRID:AB_443209), rabbit polyclonal anti-doublecortin (DCX) (1:9000, Abcam, Cat# ab18723, RRID:AB_732011), rabbit polyclonal anti-tyrosine hydroxylase (TH) (1:3000, MilliporeSigma, Cat# AB152, RRID:AB_390204), mouse monoclonal antibody anti-neuron-specific nuclear-binding protein (NeuN) (1:2000; MilliporeSigma, Cat# MAB377, RRID:AB_2298772) and rabbit polyclonal anti-glial fibrillary acidic protein (GFAP) (1:2000, MilliporeSigma, Cat# AB5804, RRID:AB_11212369). After washing three times with 0.01 M PBS, the sections were incubated with biotinylated secondary IgG antibody for 2 h at room temperature. Following washing with PBS, the sections were incubated with the ABC peroxidase for 30 min at room temperature. The peroxidase reaction was visualized with 0.05% diaminobenzidine and 0.01% hydrogen peroxide. Immunopositive cells in ipsilateral and contralateral substantia nigra compact (SNc) part were quantified in three sections of each animal. The number of immunoreactive cells for TH, NeuN, GFAP, Ki-67, and DCX was counted, respectively. Only cells with reaction products that presented within a clear border were quantified from three non-overlapping fields under a light microscope with ×200 magnification and presented as the average cell number per field on each section using ImageJ software (NIH, Bethesda, MD, United States). Data were assessed in a double-blind procedure.

### Isolation and Culture of BMSCs

BMSCs were isolated and cultured as previously described (Shen et al., 2006). Healthy specific pathogen free (SPF) grade, 3 weeks old male SD rats weighing 50–100 g were soaked in 70% ethanol for 3 min, sacrificed by cervical dislocation. Fresh bone marrow was harvested aseptically from femurs and tibias by inserting a syringe fitted with 18-gauge needle into the shaft of the bone and flushing bone marrow out with a low glucose Dulbecco’s Modified Eagle’s Medium [LG-DMEM (Gibco, Carlsbad, CA, United States)]. Then the bone marrow was mechanically dissociated by pipetting repeatedly. Next, the cells were separated from the bone marrow by a centrifuge with 1300 rpm for 3 min and incubated with complete growth medium [LG-DMEM (Gibco) containing 10% FBS (Gibco) and 1% penicillin/streptomycin (HyClone)] at 37°C in 5% CO_2_ in a sterile petri dish. After 3 days, nonadherent cells were removed by replacing the medium every three days. The adherent cells after 3–5 passages were prepared for transplantation.

### Flow Cytometry Analysis

BMSCs were analyzed for the expression of a panel of antigens. Flow cytometry analysis was performed with BMSCs at five passage of culture. Cells were incubated with fluorescent isothiocyanate (FITC)-conjugated mice anti-human CD29, CD45, and phycoerythrin (PE)-conjugated mice anti-human CD34, CD44, and CD90 (1 × 10^6^ cells/100 μL + 20 μL antibody, Becton Dickinson, Pharmingen) for phenotypic characterization. As negative controls, cells were stained with an isotype control antibody. After incubation with the antibody for 25 min, cells were washed with PBS twice and re-suspended in PBS and analyzed using flow cytometer (MoFlo XDP, Beckman Coulter, United States).

The surface antigen expression of BMSCs was identified as CD29^+^, CD44^+^, and CD90^+^, CD34^–^ and CD45^–^ (Supplementary Figure 1). The lack of CD34 and CD45 expression suggested that the cell population was depleted of hematopoietic stem cells during sub cultivation by plastic adherence. Thus, the cells used in this investigation were regarded as BMSCs.

### Transplantation of BMSCs

At 24 h after dMCAO, rats were randomly selected for BMSCs transplantation. After anesthetizing with 3–4% isoflurane in 100% oxygen, rats were injected slowly with approximately 4 × 10^6^ BMSCs in 1 mL of PBS (0.01 M, pH 7.4) or PBS only as control *via* the tail vein over a 5 min period.

### Labeling BMSCs With DiR and *in vivo*/*ex vivo* Cell Imaging

BMSCs were incubated with 10 μM DiR-PBS (PH7.4) for 30 min at 37°C according to the protocol of Xeno-Light 1, 1-dioctadecyltetramethyl indotricarbocyanine iodide (DiR) (Caliper Lifesciences). At 1 day after dMCAO, rats were injected slowly with approximately 4 × 10^6^ BMSCs-DiR^+^ (DiR-labeled) or BMSCs-DiR^–^ (DiR-nonlabeled) in 1 mL of PBS *via* the tail vein under anesthesia with 3–4% isoflurane in 100% oxygen. At 2 h, and 1, 7, 14 days after transplantation, the rats were completely anesthetized with chloral hydrate, *in vivo* their fluorescence imaging were monitored to assess bio-distribution of DiR^+^ or DiR^–^ labeled BMSCs by Xenogen IVIS Spectrum imaging system (PerkinElmer, MA, United States) under 745 nm of excitation and 800 nm of emission. Coronal brain slices, 2 mm thick, were obtained using a brain slicer for *ex vivo* imaging. Fluorescent images of each sample including cortex (−0.4 mm from bregma) and SN (−5.3 mm from bregma) were analyzed and overlaid using Living Image software (Xenogen, Alameda, CA, United States). DiR fluorescence intensity which was presented as an average radiant efficiency was plotted in units of the maximum number of photons per second per centimeter square per steradian (p/s/cm^2^/sr) (Ruan et al., 2012).

### Behavioral Testing

The behavioral tests were performed at 1, 4, 8, 12, 16, 20, 24, and 28 days after dMCAO by an investigator who was blinded to the experimental groups. All rats were familiarized with the testing environment before surgery. In the adhesive removal test, a piece of adhesive paper sized 200 mm^2^ was placed onto the contralesional forepaw (Schallert and Whishaw, 1984; Bouet et al., 2009). The rat performance was assessed by measuring the time needed sense and to remove the adhesives. The time to remove each stimulus from forelimbs was recorded on 3 trials/day. Individual trial was separated by at least 3 min. Before surgical procedure, the animals were trained 1 trial/d for 5 days. Once the rats were able to remove the tape within 2 min, they were subjected to dMCAO. In addition, neurological function was assessed by modified neurological severity scores (NSS) which is a composite of motor, sensory, reflex, and balance tests graded on a scale of 0–18 (normal score, 0; maximal deficit score, 18) (Chen et al., 2001).

### Retrograde Pseudorabies Virus Tracing

In this study, retrograde tracing were employed to label the neurons between the infarct cortex and the ipsilateral SN. The attenuated (Bartha) strain of PRV-152, which was constructed to express EGFP (a gift from Enquist L. W., Princeton University), was used. PRV-152 viruses were grown in pig kidney (PK15) cells and stored at -80°C. The final titers deters determined in PK15 cells were 1 × 10^8–9^ PFU for PRV-152 (Xu et al., 2006). Under anesthesia with 3–4% isoflurane, 3 μL PRV-152 was injected to the ipsilateral SNr of rats (5.4 mm posterior to the bregma, 2.1 mm lateral to the midline, and 8.3 mm below the dura) stereotactically at 7 days of dMCAO. The needle was left in place for an additional 30 min before it was removed. A fresh stock of virus was thawed for each injection.

### Immunofluorescence

Triple-fluorescent immunohistochemistry was performed as previously described (Zuo et al., 2018). Sections were pre-incubated with 5% normal goat serum (containing 0.2% Triton X-100) for 1 h at room temperature, and then incubated overnight at 4°C with mixtures of primary antibodies: rabbit anti-GFP (1:800, MilliporeSigma, Cat# MAB3580, RRID:AB_2313783), rabbit polyclonal anti-Ki-67 (1:1000, Abcam, Cat# ab15580, RRID:AB_443209), rabbit polyclonal anti-DCX (1:5000, Abcam, Cat# ab18723, RRID:AB_732011) and mouse monoclonal antibody anti-NeuN (1:1000; MilliporeSigma, Cat# MAB377, RRID:AB_2298772). After rinsing in 0.01 M PBS, the sections were incubated for 1 h at room temperature with the following secondary antibodies: Cy3-conjugated goat anti-mouse IgG antibody (1:100; MilliporeSigma, Cat# AP124C, RRID:AB_11213281) and FITC-conjugated goat anti-rabbit antibody (1:100; MilliporeSigma, Cat# AP307F, RRID:AB_92652). Then, sections were PBS-washed and mounted with mounting medium containing 4′, 6-diamidino-2-phenylindole (DAPI). Slides were analyzed with a confocal laser microscope (Leica Microsystems, Wetzlar, Hessen, Germany).

### Determination of Dopamine, 3, 4-Dihydroxyphenylacetic Acid, and Homovanillic Acid Concentrations in the Striata

Rats were anesthetized with 10% chloral hydrate (3.5 mg/kg, i.p.) and perfused transcardially with normal saline. According to previous study (Jackson-Lewis and Przedborski, 2007), the striata were removed quickly (within 30 s) from rats after dMCAO with or without BMSCs transplantation and placed immediately into ice-cold saline before being stored at -80°C for high performance liquid chromatography-tandem mass spectrometric (HPLC-MS/MS) analysis.

According to the protocol described by Man et al. (2019), the concentrations of dopamine (DA), 3, 4-dihydroxyphenylacetic acid (DOPAC), and homovanillic acid (HVA) in the striata were analyzed by HPLC-MS/MS (1290-6460C, Agilent Technologies, CA, United States) in National Analytical Center of China (NACC), Guangzhou. Briefly, the striata were extracted in 2 mL of 100% methanol by homogenizer and centrifuged at 4500 *g* for 5 min at 4°C. This process was repeated for 3 times. The supernatant was transferred to clean vials and evaporated to dryness under thermostat water bath at 80°C, and then reconstituted in 500 μL of 20% methanol, followed by thoroughly filtered through microporous membrane filters (0.22 μm) prior to analysis. Reference samples of DA, DOPAC and HVA were obtained from Sigma-Alorich (St. Louis, MO, United States). The samples were placed in an Agilent G1367A well plate autosampler and were chilled to 4°C. A 5 μL aliquot of sample was injected an InfinityLab Poroshell 120 Phenyl Hexyl column (4.6 × 50 mm, 2.7 μm column (Agilent Technologies, CA, United States) with a C18 Security Guard Cartridges (2.0 mm × 4.0 mm, Phenomenex, CA, United States). The mobile phase A was acetonitrile, and B was 0.1% formic acid aqueous solution. For DA, the initial mobile phase was consisted of mobile phase A and B (3:97, *v/v*), increased to 90% mobile phase A at 2.1 min and kept for 1.4 min. Then the mixture was reversed back to 3% mobile phase A at 3.6 min and kept for 1.4 min. For DOPAC and HVA, the initial mobile phase was consisted of mobile phase A and B (5:95, *v/v*), increased to 95% mobile phase A at 3.5 min and kept for 1.5 min. Then the mixture was reversed back to 5% mobile phase A at 5.1 min and kept for 0.9 min. Mass spectrometric analysis for DA was operated in the positive electrospray ionization (ESI) mode, while the analysis for DOPAC and HVA was in the negative ESI mode (Agilent Technologies, CA, United States). Quantification of the analyte was achieved by multiple reaction monitoring (MRM) with the transitions of *m/z* 153.9–136.9, 167–123, and 181–137 for DA, DOPAC and HVA, respectively. Data were analyzed using Agilent Mass Hunter (Agilent Technologies, CA, United States) with results in tissue expressed in ng/mg.

### Data Analyses

All compared data are expressed as mean ± SD. and analyzed using SPSS 13.0 (SPSS, Inc., Chicago, IL, United States). Statistical significance was determined by two-tailed Student’s *t*-test or one-way ANOVA, followed by LSD or Tamhane’s T2 *post hoc* test. *p* < 0.05 was considered statistically significant.

## Results

### Secondary Degeneration Was Observed in the Ipsilateral Substantia Nigra After Focal Cortical Infarction

**FIGURE 1:**
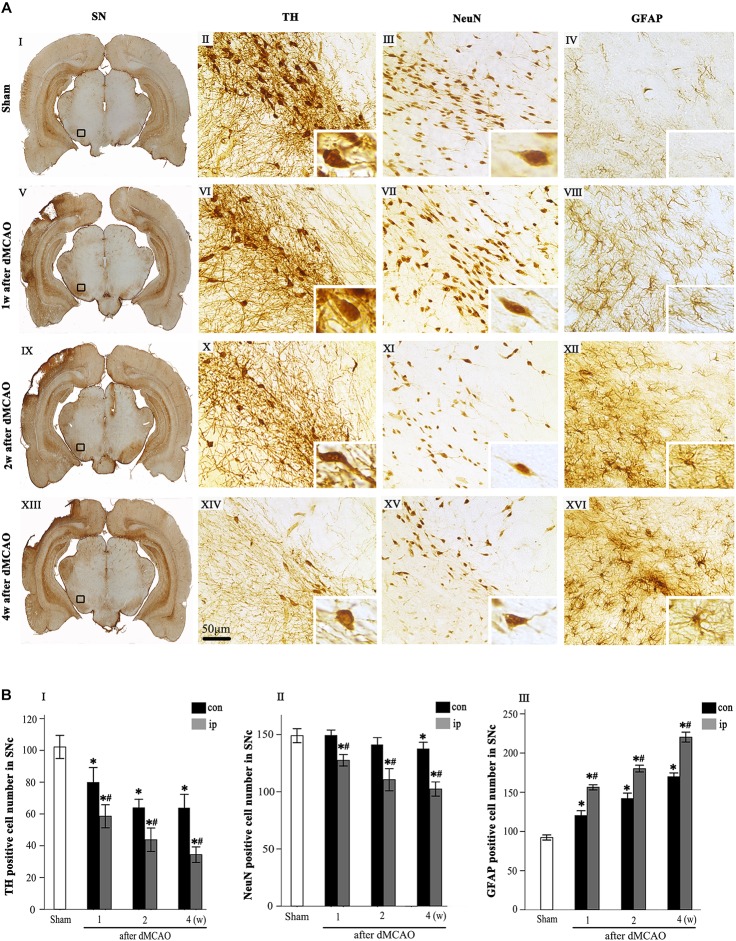
Secondary degeneration in the ipsilateral SN after dMCAO in rats. **(A)** Representative microphotographs of immunohistochemistry for TH **(II,VI,X,XIV)**, NeuN **(III,VII,XI,XV)** and GFAP **(IV,VIII,XII,XVI)** in SNc at 1 week **(V–VIII)**, 2 weeks **(IX–XII)**, and 4 weeks **(XIII–XVI)** after dMCAO and sham-operated groups **(I–IV)**. The pictures on the right are magnified from the square area on the left. Scale bar, 50 μm. **(B)** Quantitative analyses of TH^+^ **(I)**, NeuN^+^ **(II)**, and GFAP^+^ **(III)** cells in the SNc at 1, 2, and 4 weeks after dMCAO. Quantitative analyses showed that dMCAO decreased the number of TH^+^ cells in the ipsilateral [*F*_(3, 101)_ = 94.59, *p* < 0.01] and contralateral SNc [*F*_(3, 101)_ = 25.78, *p* < 0.01], and NeuN^+^ cells in the ipsilateral SNc [*F*_(3, 83)_ = 38.19, *p* < 0.01], and increased the number of GFAP^+^ cells in the ipsilateral SNc [*F*_(3, 122)_ = 638.85, *p* < 0.01] at 1, 2, and 4 weeks. Each bar represents the mean ± SD (*n* = 6 in each group). ^*^p < 0.05 vs. sham-operated group; ^#^*p* < 0.05 vs. contralateral groups at the same time point. SN, substantia nigra; SNc, substantia nigra compact part; TH, tyrosine hydroxylase; NeuN, neuron-specific nuclear-binding protein; GFAP, glial fibrillary acidic protein; Sham, sham-operated; w, week; dMCAO, distal middle cerebral artery occlusion.

Progressive neuronal damage in the ipsilateral SNc was characterized by the reduced numbers of TH^+^ and NeuN^+^ cells after dMCAO (Figure 1). Compared with the sham-operated group, the numbers of TH^+^ cells at 1, 2, and 4 weeks were significantly decreased in the ipsilateral and contralateral SNc after dMCAO (Figures 1AVI,X,XIV,BI), but this decrease in the ipsilateral SNc was more obvious. Compared to the contralateral and sham-operated groups, the number of NeuN^+^ cells in the ipsilateral SNc displayed a significant reduction after dMCAO (Figures 1AVII,IX,XV,BII). On the contrary, the number of GFAP^+^ cells in the ipsilateral SNc was increased as early as in the first week, peaking at 4 weeks after dMCAO compared with the contralateral and sham-operated groups. These astrocytes cells were characterized by their typical hypertrophic shape with thickened process (Figures 1AVIII,XII,XVI,BIII).

### Transplanted Bone Marrow Stromal Cells Migrated to the Ipsilateral Cortex and Substantia Nigra After Focal Cortical Infarction

**FIGURE 2:**
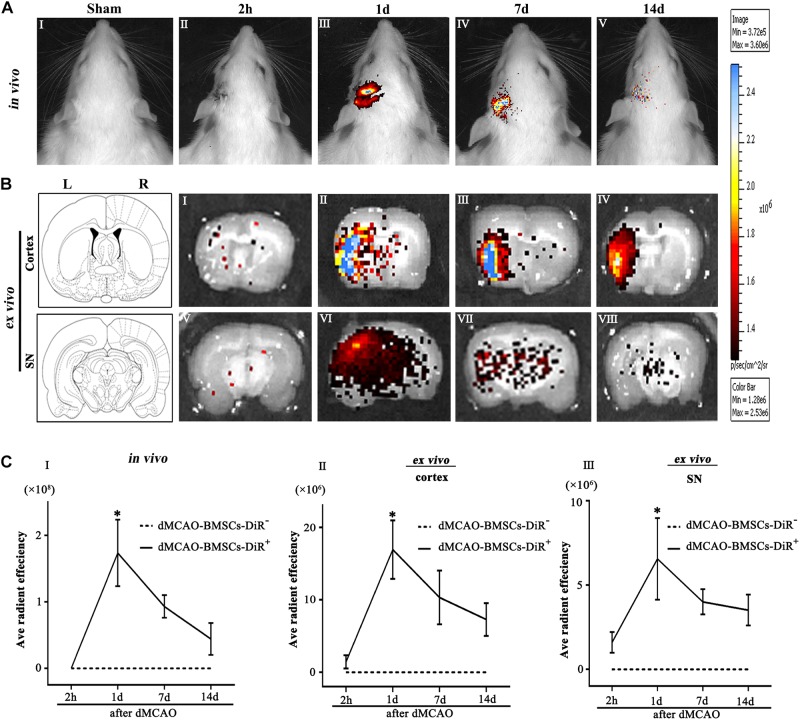
Transplanted BMSCs migrate to the ipsilateral SN after dMCAO in rats. **(A)** The *in vivo* fluorescence images of migrated BMSCs-DiR^+^ in the brain at 2 h and 1, 7, 14 days post-intravenous transplantation following focal cortical infarction. **(B)** The *ex vivo* fluorescence images of migrated BMSCs-DiR^+^ in the cortex **(I–IV)** and SN **(V–VIII)** at 2 h and 1, 7, 14 days post-intravenous transplantation following focal cortical infarction. **(C)** Quantitative analyses (*in vivo* and *ex vivo*) of migrated BMSCs-DiR^+^ in the brain **(I)**, cortex **(II)**, and SN **(III)** at 2 h and 1, 7, 14 days post-intravenous transplantation following focal cortical infarction. Compared with the sham-operated group, the fluorescence intensities of BMSCs-DiR^+^ groups both *in viv*o [*F*_(4, 25)_ = 7.67, *p* < 0.01] and *ex vivo* [cortex: *F*_(4, 25)_ = 6.58, *p* < 0.01, SN: *F*_(4, 25)_ = 4.09, *p* < 0.05] were significantly increased. ^*^p < 0.05 vs. BMSCs-DiR^–^ group (*n* = 7 in each group). Sham, sham-operated; h, hour; d, day; w, week; L, left; R, right; SN, substantia nigra; dMCAO, distal middle cerebral artery occlusion; BMSCs, bone marrow stromal cells; DiR (1,1-dioctadecyltetramethyl indotricarbocyanine iodide); DiR^+^, DiR-labeled; DiR^–^, DiR-nonlabeled.

Biodistribution of BMSCs-DiR^+^ was monitored within 14 days by IVIS imaging system. The *in viv*o fluorescence imaging showed that the transplanted BMSCs-DiR^+^ could migrate from peripheral blood to the ipsilateral brain after dMCAO (Figure 2A). The *ex vivo* fluorescence imaging demonstrated that BMSCs-DiR^+^ mainly existed in the ipsilateral cortex and SN (Figure 2B). Within the BMSCs-DiR^+^ transplanted dMCAO groups, there were no significant differences in the fluorescence intensities in the ipsilateral cortex and SN across all time points except the first day post-transplantation intravenously. No fluorescent signals were detected in the brain region of BMSCs-DiR^–^ groups both *in viv*o and *ex vivo* (Figure 2C). These results illustrated that intravenous transplanted BMSCs can migrate to the ipsilateral cortex and SN after focal cortical infarction.

### Transplanted Bone Marrow Stromal Cells Enhanced Neurogenesis in the Ipsilateral Substantia Nigra After Focal Cortical Infarction

**FIGURE 3:**
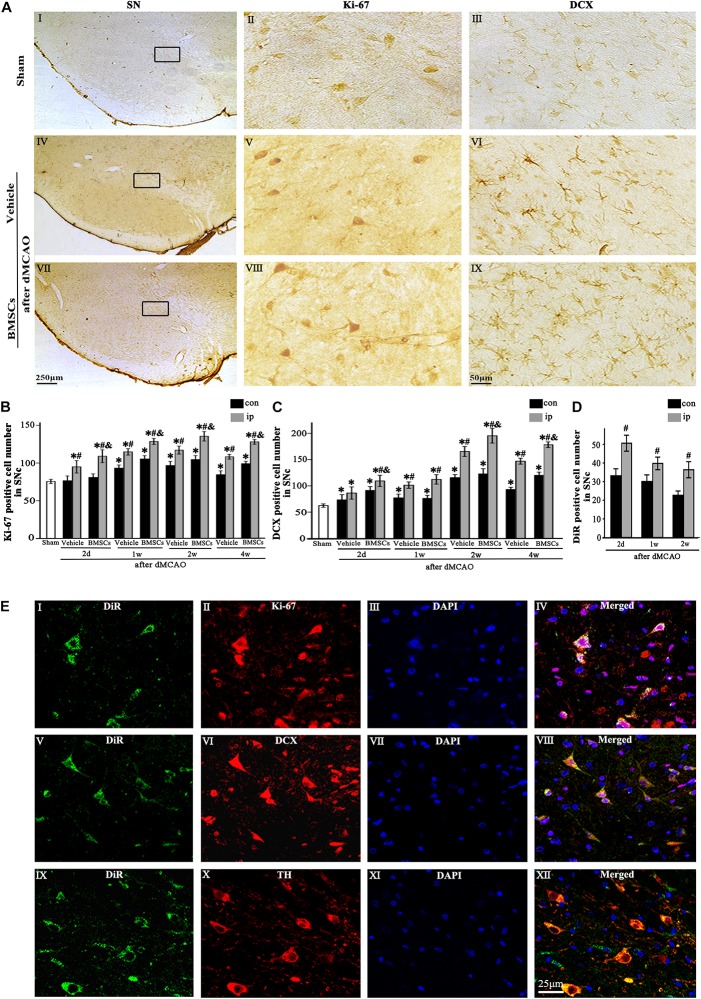
The proliferation and differentiation of BMSCs in SNc after dMCAO. **(A)** Representative microphotographs of immunohistochemistry for Ki-67 and DCX in SN after dMCAO with or without BMSCs transplantation. **(I–III)** Sham group; **(IV–VI)** vehicle group at 2 days after dMCAO; **(VII–IX)** BMSCs group at 2 days after dMCAO; **(B,C)** Quantitative analyses of Ki-67^+^ and DCX^+^ cells in SNc at 2 days, 1, 2, and 4 weeks after dMCAO. BMSCs transplantation increased the numbers of Ki-67^+^ cell [*F*_(4, 106)_ = 90.12, *p* < 0.01] and DCX^+^ cell [*F*_(4, 106)_ = 154.17, *p* < 0.01] in the ipsilateral SNc after dMCAO). **(D)** Quantitative analyses of DiR^+^ cells in SNc at 2 days, 1 and 2 weeks after dMCAO. The number of ipsilateral BMSCs-DiR^+^ cells was significantly increased after dMCAO [*F*_(2, 57)_ = 13.93, *p* < 0.01]. **(E)** Representative images of fluorescent staining of BMSCs-DiR^+^ (**I,V,IX,** green) and Ki-67 (**II**, red), DCX (**VI**, red), TH (**X**, red) and DAPI (**III,VII,XI**, blue) in SNc with BMSCs transplantation at 1 week after dMCAO. The overlapped images showed that DiR^+^ BMSCs were colocalized with Ki-67^+^, DCX^+^, and TH^+^ cells, respectively at 1 week after dMCAO **(IV,VIII,XII)**. Scale bar: A, **I, IV, VII,** 250 μm; **II, III, V, VI, VIII, IX**, 50 μm; **(E)**, 25 μm. Data are represented with mean ± S.D. ^*^p < 0.05 vs. sham-operated group, ^#^*p* < 0.05 vs. contralateral groups at the same time point and ^&^*p* < 0.05 vs. ipsilateral vehicle groups (*n* = 6 in each group). SN, substantia nigra; SNc, substantia nigra compact part; DCX, doublecortin; Sham, sham-operated; BMSCs, bone marrow stromal cells; con, contralateral; ip, ipsilateral; d, day; w, week; dMCAO, distal middle cerebral artery occlusion, DiR (1,1-dioctadecyltetramethyl indotricarbocyanine iodide), Ki-67, nuclear-associated antigen Ki-67; DAPI, 4’,6- diamidino-2-phenylindole; TH, tyrosine hydroxylase.

Immunohistochemical study showed that the Ki-67^+^ and DCX^+^ cells in sham-operated group were expressed in SNc (Figures 3AI–III), and these cells in the ipsilateral SNc stained deeply both in vehicle and BMSCs groups at 2 days after dMCAO (Figures 3AV–IX). In contrast to the sham-operated group, the number of Ki-67^+^ cell was significantly increased in the ipsilateral SNc at 2 days, and 1, 2, and 4 weeks after dMCAO with or without BMSCs transplantation, and it also increased in the contralateral SNc at 1, 2, and 4 weeks after dMCAO with or without BMSCs transplantation. Notably, BMSCs transplantation increased obviously the number of Ki-67^+^ cells in the ipsilateral SNc at 2 days, 1, 2, and 4 weeks after dMCAO when compared with vehicle groups (Figure 3B). Similarly, in comparison with the sham-operated group, the number of DCX^+^ cells in the ipsilateral SNc was increased at 2 days, 1, 2, and 4 weeks post-dMCAO. Furthermore, transplantation with BMSCs apparently increased the number of DCX^+^ cells in the ipsilateral SNc at 2 days, 2 and 4 weeks when compared with vehicle groups (Figure 3C). In comparison to the contralateral SNc, the number of ipsilateral BMSCs-DiR^+^ cells was significantly increased, with a peak at 2 days after dMCAO (Figure 3D). In addition, in the BMSCs group, the triple-labeled immunofluorescence assay showed that BMSCs-DiR^+^ cells colocalized with Ki-67^+^, DCX^+^, and TH^+^ cells in the ipsilateral SN at 1 week after dMCAO (Figures 3EI–XII). These results indicated that transplanted BMSCs could proliferate and differentiate into mature neurons in the SN after dMCAO and stimulate endogenous neurogenesis.

### Transplanted Bone Marrow Stromal Cells Improved the Neurological Functional Outcome After Focal Cortical Infarction

**FIGURE 4:**
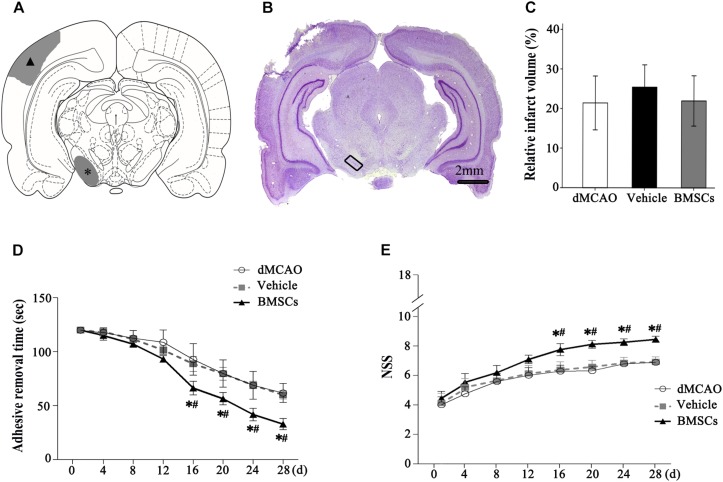
Relative infarct volume and neurological functional evaluations with or without BMSCs transplantation after dMCAO in rats. **(A)** Schematic diagram of cortical infarction (marked with arrowhead) and ipsilateral SN (marked with asterisk). **(B)** Nissl-stained coronal brain sections of focal cortical infarction at 4 weeks after dMCAO. The infarcted tissue appeared white, while the intact tissue is colored. The ipsilateral SN was marked with quadrangle. **(C)** Quantitative analyses of relative infarct volume at 4 weeks after dMCAO. There was no significant difference in the relative infarct volume among dMCAO, vehicle and BMSCs groups [*F*_(2, 18)_ = 0.478, *p* > 0.05]. **(D)** Adhesive removal test at 1–4 weeks after cortical infarction. Quantitative analyses showed that transplantation of BMSCs reduced the mean time to remove the adhesive from the forepaws after dMCAO [*F*_(2,__19) 16__*d*_ = 16.87, *p* < 0.01; *F*_(2, 19) 20__*d*_ = 19.76, *p* < 0.01; *F*_(2, 19) 24__*d*_ = 30.14, *p* < 0.01; *F*_(2, 22)__28__*d*_ = 52.75, *p* < 0.01]. **(E)** NSS at 1–4 weeks after dMCAO. Quantitative analyses showed that transplantation of BMSCs raised NSS after dMCAO [*F*_(2, 19)__16__*d*_ = 22.08, *p* < 0.01; *F*_(2, 19) 20__*d*_ = 54.54, *p* < 0.01; *F*_(2, 19) 24__*d*_ = 60.91, *p* < 0.01; *F*_(2, 18)__28__*d*_ = 79.67, *p* < 0.01]. ^*^p < 0.05 vs. dMCAO groups, ^#^*p* < 0.05 vs. vehicle groups (*n* = 7 in each group). dMCAO, distal middle cerebral artery occlusion; BMSCs, bone marrow stromal cells; NSS, neurological severity score; d, day.

The schematic diagram in Figure 4A represented that cortical infarction marked with arrowhead was induced by dMCAO and the secondary damage of ipsilateral SN marked with asterisk occurs after dMCAO. Nissl-staining assay in Figure 4B clearly showed that ischemic foci were confined within the cortex at 4 weeks after dMCAO. The relative infarction volumes at 4 weeks after dMCAO were evaluated in Figure 4C. There was no significant difference in the relative infarct volume between BMSCs and vehicle treatment groups after dMCAO. To assess the long-term functional deficits and possible recovery with or without transplanted BMSCs treatment after dMCAO, the adhesive-removal test and NSS were carried out. The mean time to remove the adhesive from the forepaws was significantly shorter in the BMSCs group than that in the vehicle group after 16 days of dMCAO (Figure 4D). Furthermore, compared with the vehicle group, rats in the BMSCs group exhibited higher NSS after 16 days of dMCAO (Figure 4E). These data suggest that BMSCs treatment could improve the neurological functional outcome after dMCAO.

### Transplanted Bone Marrow Stromal Cells Reduced Secondary Neuronal Damage in the Ipsilateral Substantia Nigra After Focal Cortical Infarction

**FIGURE 5:**
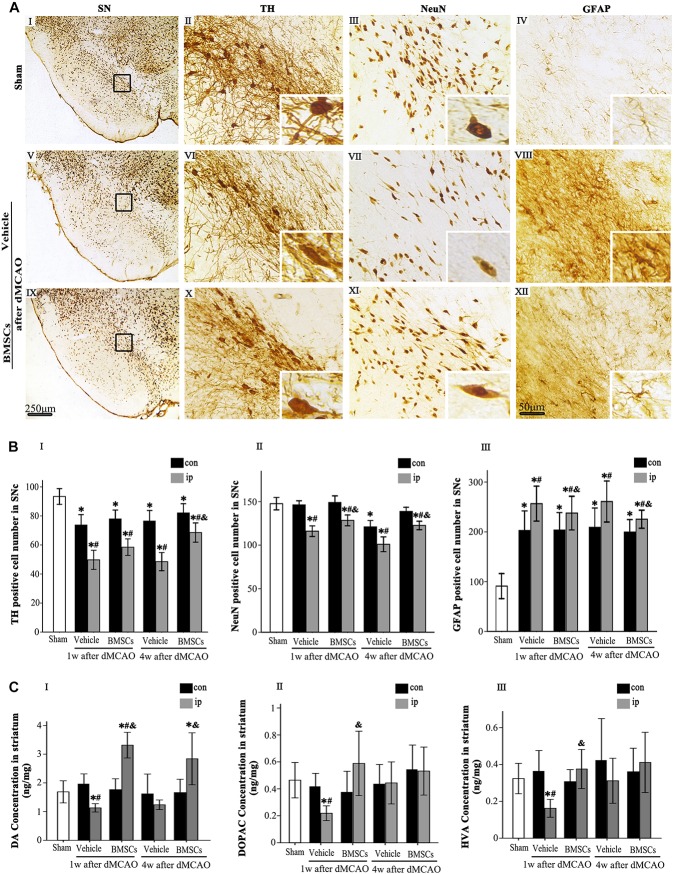
Effects of BMSCs treatment on the number of TH, NeuN, and GFAP positive cells in SN, and DA and its metabolites in striatum at 4 weeks after dMCAO. **(A)** Representative microphotographs of immunohistochemistry for TH **(II,VI,X)**, NeuN **(III,VII,XI)**, and GFAP **(IV,VIII,XII)** in SN. The pictures on the right are magnified from the square area on the left. Scale bar: **I, V, IX,** 250 μm; **II–IV, VI–VIII, X–XII,** 50 μm. **(B)** Quantitative analyses of TH, NeuN, GFAP-positive cells in the SNc at 1 and 4 weeks after dMCAO. Transplantation of BMSCs increased the numbers of TH^+^ [*F*_(2, 60)_ = 31.51, *p* < 0.01] and NeuN^+^ cells [*F*_(2, 78)_ = 17.20, *p* < 0.01], and decreased the number of GFAP^+^ cells [*F*_(2, 78)_ = 1848.10, *p* < 0.01] in the ipsilateral SNc after dMCAO. Each bar represents the mean ± S.D. ^*^p < 0.05 vs. sham-operated group, ^#^*p* < 0.05 vs. contralateral groups at the same time point and ^&^*p* < 0.05 vs. ipsilateral vehicle groups (*n* = 6 in each group). **(C)** The DA **(I)**, DOPAC **(II)**, and HVA **(III)** concentrations in striata at 1 and 4 weeks after dMCAO with or without BMSCs transplantation. Transplantation with BMSCs increased the concentration of DA [*F*_(2, 8)_ = 6.33, *p* < 0.05] in the ipsilateral striatum after dMCAO, maintained the concentration of DOPAC [*F*_(2, 11)_ = 0.53, *p* > 0.05] and HVA [*F*_(2,__11)_ = 0.67, *p* > 0.05] in the ipsilateral striatum after dMCAO. Each bar represents the mean ± SD ^*^p < 0.05 vs. sham-operated group and *^#^p* < 0.05 vs. contralateral groups at the same time point and *^&^p* < 0.05 vs. ipsilateral vehicle groups (*n* = 4 in each group). SN, substantia nigra; SNc, substantia nigra compact part; TH, tyrosine hydroxylase; NeuN, neuron-specific nuclear-binding protein; GFAP, Glial fibrillary acidic protein; Sham, sham-operated; BMSCs, bone marrow stromal cells; DA, dopamine; DOPAC, 3,4-dihydroxyphenylacetic acid; HVA, homovanillic acid; con, contralateral; ip, ipsilateral; w, week; dMCAO, distal middle cerebral artery occlusion.

To determine whether transplanted BMSCs offer neuroprotection in the ipsilateral SN after focal cortical infarction, neuronal damage and astrocytic activation were evaluated in the SNc at 1 and 4 weeks after dMCAO, respectively. Compared with sham-operated and contralateral groups, the numbers of TH^+^ and NeuN^+^ cells were significantly decreased, while GFAP^+^ cells were increased in the ipsilateral SNc at 1 and 4 weeks after dMCAO. As expected, after BMSCs treatment, the numbers of TH^+^ cell at 4 weeks and NeuN^+^ cell at 1 and 4 weeks were evidently increased in the ipsilateral SNc and GFAP^+^ cell were decreased at 1 and 4 weeks after dMCAO when compared to vehicle groups (Figures 5A,B). Furthermore, we detected the concentrations of DA and its metabolites, DOPAC and HVA, in the striata at 1 and 4 weeks after dMCAO with or without BMSCs treatment. Compared with sham-operated and contralateral groups, the concentration of DA was significantly decreased in the ipsilateral striatum at 1 week after dMCAO. Transplantation of BMSCs apparently increased the concentration of DA in the ipsilateral striata at 1 and 4 weeks after dMCAO (Figure 5CI). Similarly, the concentrations of DOAPC and HVA were significantly decreased in the ipsilateral striatum at 1 week after dMCAO when compared with sham-operated and contralateral groups. As expected, transplantation of BMSCs prevented the decline of the concentration of DOAPC and HVA in the ipsilateral striatum at 1 week after dMCAO (Figures 5CII,III). These data showed that BMSCs transplantation could mitigate the secondary damage through promoting dopaminergic neuronal survival and alleviating astrocytic activation, thereby increasing contents of DA and its metabolites, in the ipsilateral SN after dMCAO.

### Transplanted Bone Marrow Stromal Cells Enhanced the Cortico-Striatum-Nigral Connections After Focal Cortical Infarction

**FIGURE 6:**
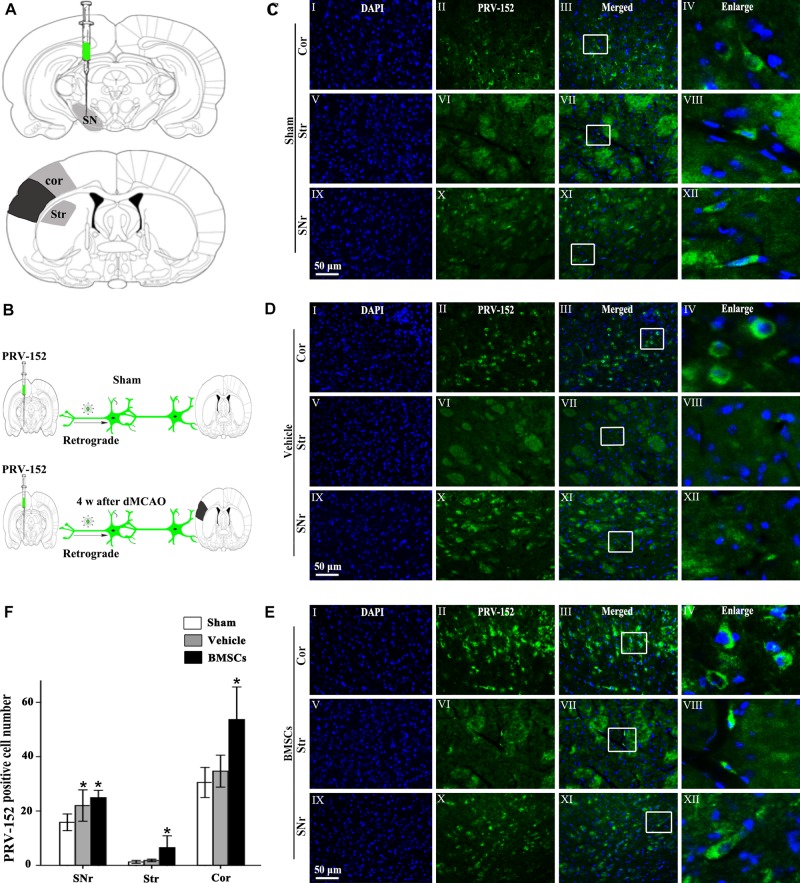
Cortico-striatum-nigral tract retrograde tracing with PRV-152. **(A)** PRV-152 was injected into the ipsilateral SNr. Regions of interest (ROI) of PRV-152 positive cell counting were shown. Dark shaded area represents ischemic region after dMCAO; lighter shaded area represents ROI. **(B)** Schematic illustration of the fluorescent signal of PRV-152 in sham-operated and dMCAO groups with or without BMSCs transplantation after PRV-152 was injected into the ipsilateral SNr. **(C)** Representative photographs of fluorescent double staining of PRV-152 (green) and DAPI (blue) in the ipsilateral cortex **(I–IV)**, striatum **(V–VIII)** and SNr **(IX-XII)** at 4 days after PRV-152 injection in sham-operated group. **(D)** Representative photographs of fluorescent double staining of PRV-152 (green) and DAPI (blue) in the ipsilateral cortex **(I–IV)**, striatum **(V–VIII)**, and SNr **(IX–XII)** at 4 weeks after PRV-152 injection in vehicle group. **(E)** Representative photographs of fluorescent double staining of PRV-152 (green) and DAPI (blue) in the ipsilateral cortex **(I–IV)**, striatum **(V–VIII)** and SNr **(IX–XII)** at 4 weeks after PRV-152 injection in BMSCs group. Scale bar, 50 μm. **(F)** Quantitative analyses of PRV-152^+^ cell number in the ipsilateral cortex, striatum and SNr after dMCAO. Transplantation with BMSCs increased PRV-152^+^ cells in the ipsilateral cortex [*F*_(2, 19)_ = 9.85, *p <* 0.01], striatum [*F*_(2,__41)_ = 10.67, *p <* 0.01] and SNr [*F*_(2,__19)_ = 6.05, *p <* 0.01] after dMCAO. Each bar represents the mean ± SD ^*^p < 0.05 vs. sham-operated group and ^#^*p* < 0.05 vs. vehicle group (*n* = 7 in each group). SNr, substantia nigra pars reticulata; Cor, cortex; Str, striatum; PRV, pseudorabies virus; Sham, sham-operated; w, week; dMCAO, distal middle cerebral artery occlusion; BMSCs, bone marrow stromal cells; DAPI, 4’, 6- diamidino-2-phenylindole.

To investigate whether BMSCs transplantation enhanced cortico-striatum-nigral connection after dMCAO, PRV-152 for retrograde tracing was injected stereotaxically to the ipsilateral SN with or without BMSCs transplantation at 7 days of dMCAO (Figure 6). Figure 6A is the schematic diagram showed that PRV-152 was injected to the ipsilateral SNr of rats, and the regions of interest of cortex, striatum and SNr. After PRV-152 was injected into the ipsilateral SNr, PRV-152 would reach to the regions of interest aforementioned by the way of retrograde axoplasmic transport in sham-operated and dMCAO groups (Figure 6B). PRV-152^+^ staining in the ipsilateral cortex, striatum and SNr were detected by immunofluorescence assay. Figures 6C–E showed that PRV-152^+^ cells were characterized by PRV-152^+^ staining around the nucleus (Figures 6C–E). In sham-operated rats, few PRV-152^+^ cells were visible in the ipsilateral cortex, striatum and SNr (Figures 6CI–XII). In the vehicle group, PRV-152^+^ cells mainly distributed in the ipsilateral cortex, at 4 weeks after focal cortical infarction (Figures 6DI–XII). Importantly, compared with sham-operated and vehicle groups, the number of PRV-152^+^ cells in BMSCs treatment group was obviously increased in the ipsilateral cortex, striatum and SNr at 4 weeks after focal cortical infarction (Figures 6EI–XII,F). These results suggested that the intravenous transplantation of BMSCs potentially enhanced cortico-striatum-nigral connections after dMCAO.

## Discussion

In this study, we confirmed that transplanted BMSCs intravenously could migrate to the ipsilateral SN in adult SD rats after dMCAO. BMSCs transplantation significantly improved neurological functional outcome and attenuated the secondary nigral degeneration following focal cerebral ischemia. In addition, BMSCs enhanced neurogenesis in the ipsilateral SN and cortico-striatum-nigral connections after focal cortical infarction. To our knowledge, this is the first report that BMSCs transplantation exerts neuroprotective effects against the secondary damage in the SN after focal cerebral infarction.

After focal cerebral infarction in the dMCAO territory, neuronal death, axonal degeneration, gliosis and marked atrophy have been found in the ipsilateral thalamus, SN, distal pyramidal tract and so on (Tamura et al., 1990; Zhang et al., 2012). Being not directly affected by primary ischemic lesion, the midbrain SN has been a suitable site to study remote lesion after cortical ischemic stroke. SN is densely composed of dopaminergic neurons. As a marker of dopaminergic neurons, TH was employed to investigate neuronal loss within the region in this study. We found the obvious losses of nigral TH^+^ and NeuN^+^ neuron and gliosis in the ipsilateral SNc after dMCAO. Accordingly, the concentration of DA and its metabolites (DOPAC and HVA) in the ipsilateral striatal tissue was decreased at 1 week after dMCAO. It is known that the nigrostriatal pathway arises from SNc DA neurons that primarily project their axons to the striatum and release DA to control motor activity (Gower and Tiberi, 2018). Our results could be interpreted as retrograde, anterograde and transsynaptic reactions in the ipsilateral SN to partial deafferentation after stroke (Tamura et al., 1990; Uchida et al., 2010; Yang et al., 2014). Owing to progressive secondary degeneration will occur at several days or weeks after focal cerebral infarction, it is possible to be a therapeutic target beyond the narrow time window for acute stroke (Prinz et al., 2015).

The advances in BMSCs biology have offered a number of enticing potential avenues for the treatment of multiple diseases including ischemic stroke. In the present study, BMSCs were uniformly negative for the hematopoietic lineage marker CD34 and the leukocyte common antigen CD45 but positive for CD29, CD44, and CD90, which are routinely used to characterize the MSCs (Zhao et al., 2016). These CD29^+^, CD44^+^, and CD90^+^ BMSCs displayed highly proliferative potential as reported previously (Hou et al., 2007; Xue et al., 2015). Therefore, they could be easily expanded to a sufficient amount for transplantation. As evidenced by biodistrubution imaging analyses *in vivo* and *ex vivo*, BMSCs-DiR^+^ can selectively migrate from peripheral blood to the ipsilateral cortex and SN within 14 days after dMCAO. It represents that BMSC migrated to brain. Over time, the number of BMSCs-DiR^+^ were gradually decreased, which may due to the effect of fluorescent quenching of DiR (Cho et al., 2012), but Ki-67^+^ (as a marker of proliferation) and DCX^+^ (as a marker of differentiation) cells were significantly increased from second to 28th day after dMCAO, suggesting that transplanted BMSCs stimulated endogenous nigral neurogenesis, and then exerted neurorestorative effects. Multiline age differentiation potential of BMSCs can differentiate into ectodermal-lineage cells, neurons and neuroglia (Jiang et al., 2002; Bosnakovski et al., 2005). Interestingly, BMSCs can spontaneously indicate some neuronal markers, such as DCX and TH in the SN at 7 days after dMCAO with immunofluorescent staining of DiR^+^/DCX^+^ and DiR^+^/TH^+^ cells. It is evident that after transplantation of BMSCs, the number of TH^+^ cell was increased at 28 days post-ischemia, suggesting that the transplanted BMSCs can differentiate into dopaminergic neurons or promote the survival of the remaining dopaminergic neurons in the SN, thereby increasing concentration of DA and preventing the decline of DOAPC and HVA in the ipsilateral striatum at 1 week after dMCAO.

It is accepted that progenitor cells with neurogenic potential reside in the adult SN and can give rise to new neurons when exposed to appropriate environmental signals (Lie et al., 2002). On the other hand, transplanted BMSCs reduce neuronal apoptosis and promote neuronal proliferation through releasing of neurotrophins, growth factors and other supportive substances (Kinnaird et al., 2004; Qu et al., 2007; Deng et al., 2010; Wakabayashi et al., 2010; Shichinohe et al., 2015), increasing axonal sprouting and promoting axonal plasticity (van Velthoven et al., 2012) after stroke. In addition, the immunomodulatory function is also considered to account for the beneficial effects of BMSCs in stroke rats (Ohtaki et al., 2008; Pavlichenko et al., 2008; Tsai et al., 2014; Tan et al., 2018). Therefore, engrafted BMSCs may provide an appropriate environment to reduce neuronal damage and promote neurogenesis in the SN after cerebral ischemia. Our current study showed, although the relative infarct volume in the BMSCs transplanted rats at 28 days post-ischemic stroke did not significantly differ from the vehicle group, that the increase of neuron, decrease of astrocyte and improvement of behavioral functional outcome implied that intravenous transplantation of BMSC exerts neuroprotective role through alleviating secondary injuries in the SN after cortical infarction.

To date, the underlying mechanisms of secondary degeneration in remote sites after ischemic stroke have not been completely clarified. Previously, we have demonstrated the occurrence of secondary degeneration in the ipsilateral thalamus and SN, and the impairment of cortical-striatum-nigral connections after cortical infarction (Zuo et al., 2016, 2018), suggesting the anterograde degeneration, retrograde degeneration, and transneuronal degeneration might be the major mechanism. It is possible that BMSCs transplantation improves neurofunctional restoration through increasing axonal sprouting, upregulating the expression of synaptophysin in corticospinal and corticorubral tracts and neural/glial antigen 2 in white matter after focal cerebral ischemia (Liu et al., 2007; Yu et al., 2018). BMSCs can promote axonal plasticity and remyelination after stroke, which conduce to preserve interhemispheric cortical connections (Nagahama et al., 2018), reinforce inter- and intracortical axonal connections (Liu et al., 2010) and restore thalamocortical circuits (Song et al., 2013). In current study, transplanted BMSCs enhanced the cortico-striatum-nigral connections after focal cortical infarction might be related to the induction of axon and myelin remodeling.

In summary, our study provides the compelling evidence that intravenous transplanted BMSCs could migrate to the ipsilateral SN and cortex after focal cortical infarction. The proliferation and differentiation of BMSCs stimulates endogenous neurogenesis. In addition, we demonstrated that BMSCs transplantation exerts neurorestorative effects through alleviating the secondary damage of dopaminergic neuron, enhancing cortico-striatum-nigral connections, increasing contents of DA and its metabolites, reducing gliosis, and improving the neurological functional outcome after focal cortical infarction. These evidences provide a promising therapeutic strategy for repairing post-dMCAO secondary damage in the ipsilateral SN.

## Ethics Statement

This study was carried out in accordance with the recommendations of Animal Research: Reporting *in vivo* Experiments guidelines. The protocol was approved by the Animal Care and Use Committee of Guangzhou Medical University (Guangzhou, China).

## Author Contributions

EX and JJ designed the experiments. JJ and YT collected the data. JJ performed the experiments with assistance from YT and XZ. WS performed the experiments related to the fluorescent staining of immunohistochemistry. The manuscript was prepared by EX with assistance from JJ, YT, KL, and LZ. All authors read and commented on the manuscript, and approved the final version of the manuscript.

## Conflict of Interest Statement

The authors declare that the research was conducted in the absence of any commercial or financial relationships that could be construed as a potential conflict of interest.

## Abbreviations

BMSCs: bone marrow stromal cells
con: contralateral
Cor: cotrex
d: day
DA: dopamine
DAPI: 4′,6-diamidino-2-phenylindole
DCX: doublecortin
DiR^+^: DiR-labeled
DiR: 1,1-dioctadecyltetramethyl indotricarbocyanine iodide
DiR^–^: DiR-nonlabeled
dMCAO: distal middle cerebral artery occlusion
DOPAC: 3,4-dihydroxyphenylacetic acid
ESI: electrospray ionization
GFAP: glial fibrillary acidic protein
HPLC-MS/MS: high performance liquid chromatography-tandem mass spectrometric
HVA: homovanillic acid
ip: ipsilateral
Ki-67: nuclear-associated antigen Ki-67
MRM: multiple reaction monitoring
NeuN: neuron-specific nuclear-binding protein
NSS: neurological severity score
PRV: pseudorabies virus
Sham: sham-operated
SN: substantia nigra
SNc: substantia nigra compact par
SNr: substantia nigra pars reticulata
Str: striatum
TH: tyrosine hydroxylase
w: week.
