# Supplementary material for: Bone Marrow Stromal Cells Alleviate Secondary Damage in the Substantia Nigra After Focal Cerebral Infarction in Rats
Source: Front Cell Neurosci. 2019 Jul 24;13:338. doi: 10.3389/fncel.2019.00338 (PMC6668054; doi:10.3389/fncel.2019.00338)
Supplement: Supplementary file 1 [file Table_1.DOCX]

Supplementary Material

## Supplementary Figure


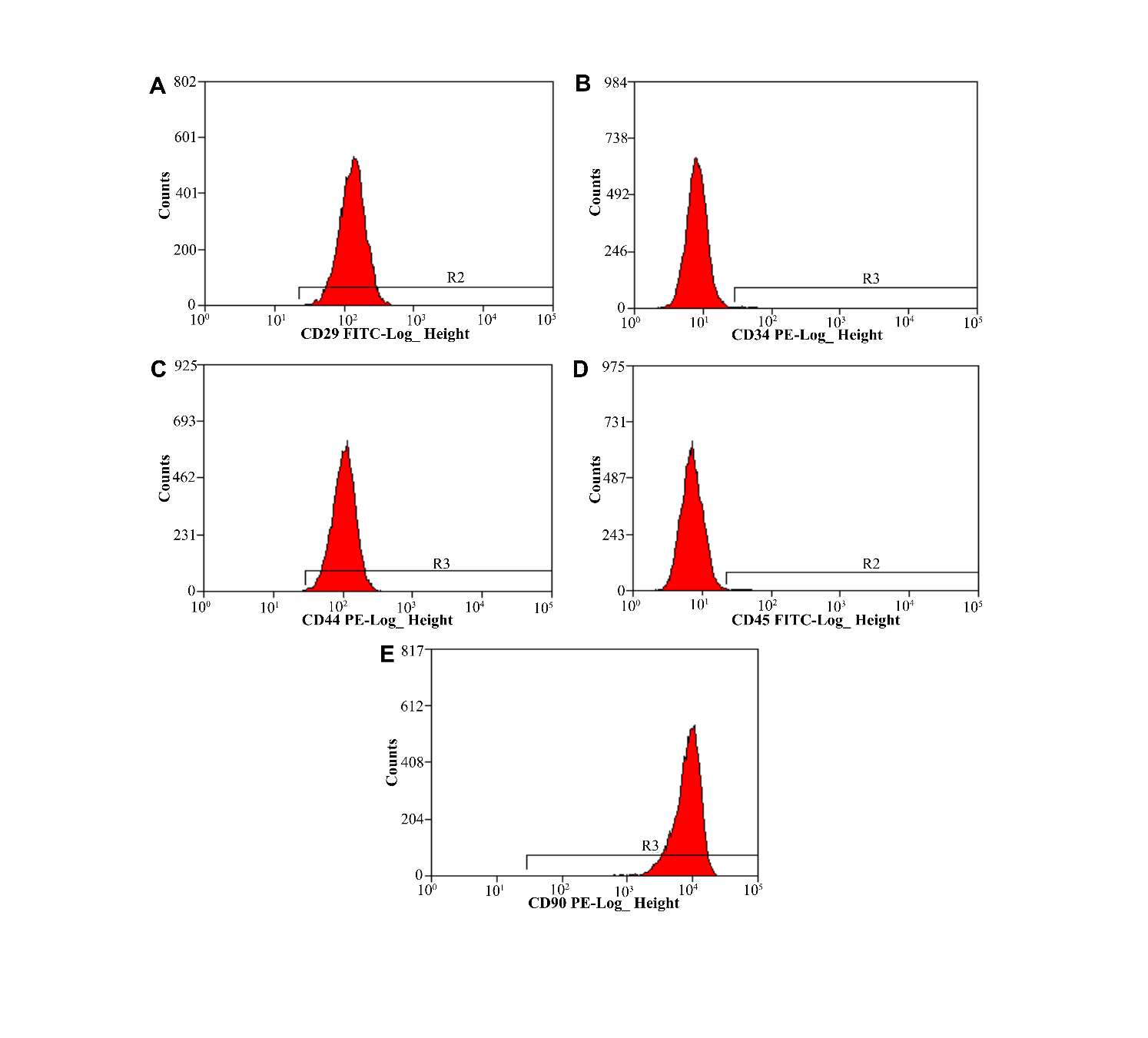


**Supplementary Figure.** Characterization of bone marrow stromal cells isolated from rats. Cells were harvested from rats and adherent mesenchymal cells were isolated. (A-E) Rat’s BMSCs were identified by fluorescence-activated cell sorting with a FACSCalibur cytometer, confirming positive expression of CD29, CD44 and CD90, and negative identification of CD34 and CD45.
